# Challenges and Prospects of Patient-Derived Xenografts for Cancer Research

**DOI:** 10.3390/cancers15174352

**Published:** 2023-08-31

**Authors:** Jiankang Jin, Katsuhiro Yoshimura, Matheus Sewastjanow-Silva, Shumei Song, Jaffer A. Ajani

**Affiliations:** Department of Gastrointestinal Medical Oncology, The University of Texas MD Anderson Cancer Center, Houston, TX 77030, USA; jjin@mdanderson.org (J.J.); kyoshimura@mdanderson.org (K.Y.); msewastjanow@mdanderson.org (M.S.-S.)

**Keywords:** patient-derived xenograft (PDX), orthotopic PDX (PODX), humanized mice (HM), tumor microenvironment (TME), intronic quantitative PCR (qPCR), authentication and quantification of biosamples

## Abstract

**Simple Summary:**

The patient-derived xenograft (PDX) model is the in vivo standard for cancer research as a preclinical platform. Besides its merits, we describe the general rationale of various PDX models and the standard procedures of orthotopic models of gastric adenocarcinoma. We also present challenges with these models, such as loss of heterogeneity, selection bias, clonal evolution, unsuitability for immune-oncology studies, viral infections, host stroma contaminations, and oncogenic transformation of host cells, among others. Additionally, we present the emerging research prospects, such as using 3D organoid culture, humanized mouse models, syngeneic mouse models to improve interrogations, and an intronic qPCR method of biosample authentication and quantification.

**Abstract:**

We discuss the importance of the in vivo models in elucidating cancer biology, focusing on the patient-derived xenograft (PDX) models, which are classic and standard functional in vivo platforms for preclinical evaluation. We provide an overview of the most representative models, including cell-derived xenografts (CDX), tumor and metastatic cell-derived xenografts, and PDX models utilizing humanized mice (HM). The orthotopic models, which could reproduce the cancer environment and its progression, similar to human tumors, are particularly common. The standard procedures and rationales of gastric adenocarcinoma (GAC) orthotopic models are addressed. Despite the significant advantages of the PDX models, such as recapitulating key features of human tumors and enabling drug testing in the in vivo context, some challenges must be acknowledged, including loss of heterogeneity, selection bias, clonal evolution, stroma replacement, tumor micro-environment (TME) changes, host cell carryover and contaminations, human-to-host cell oncogenic transformation, human and host viral infections, as well as limitations for immunologic research. To compensate for these limitations, other mouse models, such as syngeneic and humanized mouse models, are currently utilized. Overall, the PDX models represent a powerful tool in cancer research, providing critical insights into tumor biology and potential therapeutic targets, but their limitations and challenges must be carefully considered for their effective use. Lastly, we present an intronic quantitative PCR (qPCR) method to authenticate, detect, and quantify human/murine cells in cell lines and PDX samples.

## 1. Introduction

Cell cultures and in vivo mouse models are the commonly used methods in cancer research. The cell lines are from the in vitro model, primarily used in basic cancer research and drug discovery, providing an indefinite source of biological material for experimental purposes. Cancer cell lines retain many genetic properties of the cancers of origin [1]. The patient-derived xenograft (PDX) model, as a representative of in vivo models, is popular as it allows for the direct assessment of tumor properties using patient specimens. The PDX models are commonly established through the subcutaneous injection of tumor cells, regardless of their origin (heterotopic) or engraftment in the corresponding organs (orthotopic) [2]. This model offers direct means of addressing clinically relevant questions, such as drug screening and evaluating the efficacy of drugs. The PDX models also allow for the study of evolutionary cancer dynamics during progression and drug exposure, as well as the underlying mechanisms of resistance. Although the ability of the PDX models to predict clinical outcomes is not accurate, the addition of new measures, such as the humanized mouse models, can improve predictions and aid in therapeutic decisions. PDXs can also recapitulate the malignant characteristics of different tumors from different patients [3]. In this review, we discuss and summarize the functional roles of the PDX models in cancer research, with an emphasis on the challenges that this model and related mouse models face. Additionally, we introduce the standard procedure and general characteristic of the solid orthotopic mouse model of GAC. We also introduce a novel intronic genomic qPCR to quantify and authenticate human and murine cell lines, as well as PDX tumors. Finally, we explore future perspectives in cancer research by using the PDX and other xenograft models. In this review, we extract findings on all cancer types, draw conclusions in each section, and then, exemplify major points using GAC illustrated in figures.

## 2. Overview of PDXs

### 2.1. PDX as the Standard in vivo Model for Cancer Research

The PDX models are the standard platform for translational cancer research, drug screening, and treatment, biomarker development, preclinical evaluation of personalized medicine strategies, and personalized cancer therapy [4]. In 2019, The National Cancer Institute launched a national repository of patient-derived models, including PDXs and in vitro patient-derived cell cultures (https://dtp.cancer.gov/repositories/) (accessed on 23 August 2023). In 2023, the European Molecular Biology Laboratory and the Jackson Laboratory also launched a platform called PDCM Finder (https://www.cancermodels.org) (accessed on 23 August 2023) for patient-derived cancer models (PDCMs). It aggregates clinical, genomic, and functional data obtained from PDXs, organoids, and cell lines. The platform standardized and integrated over 90 million data points from more than 4500 PDX models [5].

The cell-derived xenograft (CDX) and PDX models are popular rodent (typically mice or rats) models for studying human cancers. To create the CDX models, human cancer cell lines are injected into T-cell-deficient nude or severe combined immunodeficient (SCID) mice. In the CDX models, the cells from established cell lines were derived from cancer patients, such as AGS, GT5, KatoIII, MKN45, Snu16, etc., in the context of GAC [6]. For the PDX models, patient-derived tumor fragments, metastasized cells/tissue, circulating free cells, patient-derived malignant ascites, or cancer cells briefly going through in vitro expansion that are xenografted into rodents are termed as the PDX models in a broad sense (PDX sensu lato); however, the PDX models sensu stricto (i.e., in a narrow sense) only include patient-derived tumor fragments and metastasized tissues. Compared with the CDX models, PDX models are more relevant to human cancer biology and are better suited for drug screening, but they are not ideal [7]. A significant characteristic of PDXs sensu stricto is that they are directly implanted into mice without intermediate cell culture, which introduces more complexities. In this review, we use the term PDX models in a broad sense, including not only PDXs that are strictly defined but also CDXs, patient-derived orthotopic xenografts (PDOXs), and other models that involve xenografting patient-derived cells, tissues, and tumors into mouse models.

Tumor fragments or metastasized cells, such as those from malignant ascites cells, are commonly used to create the PDX models by surgically transplanting them into immunodeficient mice. The susceptibility of these models to therapeutic drugs may be closely correlated with data in patients. These models can closely mimic the patient’s tumor, and they are highly useful in predicting the efficacy of drugs. PDXs also allow a “co-clinical trial” approach, where pre-clinical investigations in vivo and clinical trials can be performed, in parallel or sequentially, to assess drug efficacy [8]. Validation studies have shown that the PDX models, mostly, have identical mutational profiles to patient tumors and provide them for drug screening [9]. The PDX models are considered better than cell culture models in recapitulating the histological features and molecular characteristics [10]. For example, in preclinical chemotherapy for breast cancer, 113 tumors were implanted to form PDXs with an overall take rate of 27.4%, and PDXs with the same molecular subtype as the patients were observed in 28 (90.3%) of 31 cases [11]. It was previously shown that PDXs grown in immunodeficient mice closely resemble the original tumors both histologically and genetically [12].

Kopetz et al. (2012) compared PDXs, cancer cells in vitro, and CDXs, and found that PDXs retained key characteristics of tumors from patients, including histologic characteristics, genomic signatures, and the heterogeneity of cancer cells. PDXs even retained stromal and immune cells originating from the patients, making them a more precise model for reproducing the in vivo environment to test drug response [13]. In GAC, Cho et al. (2016) showed good performance of PDXs by testing the combination of the BCL2L1 inhibitor and a cytotoxic drug in *BCL2L1*-amplified tumors. They observed promising in vivo drug efficacy with significant tumor shrinkage [14]. However, to achieve precision in vivo studies and preclinical testing, PDX tissue banking by cryopreservation has become indispensable to maintain clinical samples’ heterogeneity, vitality, and genetic makeup.

### 2.2. Types of PDX Models

Regarding types of PDXs, two types of PDX engraftment are discussed, including heterotopic and orthotopic models. Heterotopic models involve subcutaneous implantation and other subtypes, such as peritoneal injection and tail vein injection. On the other hand, the orthotopic model, the PDOX, involves placing engraftments in the corresponding organs to those in the primary tumors.

Previous reviews have classified PDXs into eight types based on the biomaterial for implantation and the background of the experiment [3,15,16]. Byrne et al. (2017) [3] detailed the eight types of PDXs: the three common PDX types in Table 1 plus the other five less common types of PDXs: ref. [4] metastatic tumor specimens implanted orthotopically at the metastatic site, ref. [5] metastatic tumor specimens implanted subcutaneously, ref. [6] minimal residual disease, ref. [7] clinical trial-associated xenografts, and [8] circulating tumor cell (CTC)-derived PDXs.

Furthermore, PDX was defined as one of six animal models, and it listed retaining heterogeneity and mutations, tumor microenvironment (TME), intact endocrine system, metastasis assessment, and tumor biobank formation as advantages of PDXs [17]. However, all PDX models have limitations, such as being generated in mice with deficient immunity, having different tumorigenesis, and being unsuitable for early-stage cancer, as elaborated later in this paper.

**Figure 1:**
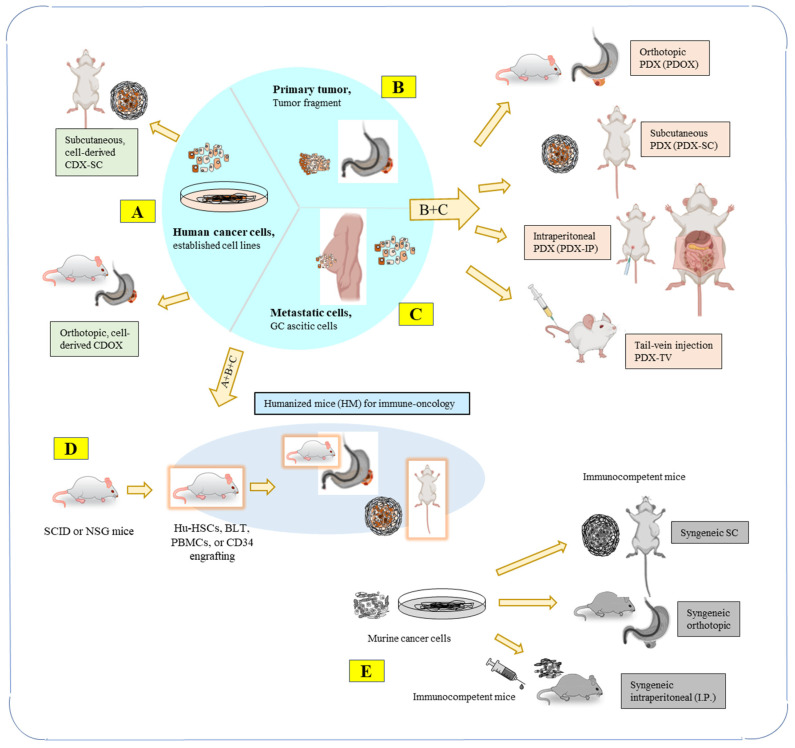
Types of PDX models for cancer research are illustrated using gastric adenocarcinoma. (GAC) cells as examples: (**A**) Human cancer cells derived from cell lines are used to establish mouse models of CDX-SC and CDOX; (**B**) primary tumors from patients are used directly for engraftment; (**C**) metastatic cells, such as GAC ascitic cells, are concentrated and engrafted. From (**A**–**C**), mouse models of PDX, PDOX, PDX-IP, and PDX-TV are commonly established. (**D**) Humanized mice (HM) models (see Section 4.2) for immune-oncology studies, human derived Hu-HSCs (hematopoietic stem cells), BLT (bone marrow, liver, and thymus), PBMCs (human peripheral blood mononuclear cells), or CD34+ cells are engrafted into immuno-incompetent mice, such as SCID or NSG mice. Immune cells, such as T cells, are preferably educated in the human system, simultaneously or subsequently. Cancer cells are, then, engrafted to create HM-PDX-SC or HM-PDOX models. (**E**) Syngeneic mouse models (see Section 4.3) (non-PDX models) use immunocompetent mice, and engraftments are from murine cell lines, administered through subcutaneous (S.C.), intraperitoneal (I.P.), or orthotopic injections. Mouse models in (**D**,**E**) are discussed in detail in Prospects Section 4.2 and Section 4.3.

An illustration of commonly used PDX using GAC primaries and metastatic cells, such as those from malignant ascites, cancer-derived cell lines, and humanized mouse (HM) models, together with syngeneic mouse models, as shown in Figure 1.

### 2.3. Mouse Host Types for PDX Model

Various types of immunodeficient mice are used in PDX models. It is important to understand the specific immunodeficiency characteristics of each mouse strain. We have summarized the features of the main immunodeficient mouse models used in PDX models in Table 2. The commonly used mouse strains in PDX models include athymic nude mice (Foxn1 null), Rag1/Rag2 mice (Rag1/2 recombinase defects), SCID mice (mutated Prkdc gene), SCID/Beige mice (combined mutated Beige with SCID), NOD/SCID mice (NOD, non-obese diabetic mutation with SCID), NOG and NSG mice (NOD/SCID plus IL2Rγ truncation), as well as NRG mice (NOG with Rag1 mutation, replacing SCID mutation) [17,18]. Cho et al. (2016) listed, in their review, the status of immune cells, such as mature B, mature T, dendritic cells, macrophages, and natural killer (NK) cells in NSG, NOD-SCID, BALB-SCID, B6 Rag1, and nude mice [14].

### 2.4. Orthotopic PDX Models (PDOXs) as an Emerging Trend

Regarding GAC PDOXs, 10 studies were identified, of which 70% were CDXs. In 90% of these studies, implantation was performed in the subserosal layer of the stomach wall. Tumor engraftment success rates varied widely, ranging from 0 to 100%. In studies utilizing either cell suspension or tumor fragments, metastases were observed in 40% of PDOXs implanted into the subserosal layer. However, there is insufficient evidence to determine whether the submucosal site is more effective than the subserosal layer or whether tissue fragments are more successful than cell suspensions for engraftment and metastases. Our group has extensively utilized PDOXs for GAC research, and our models have demonstrated high success rates and reproducibility. Here, we illustrate our standard protocol and results for PDOX using a murine cell line: GFP/luciferase-labeled KP-Luc2 cells.

**Figure 2:**
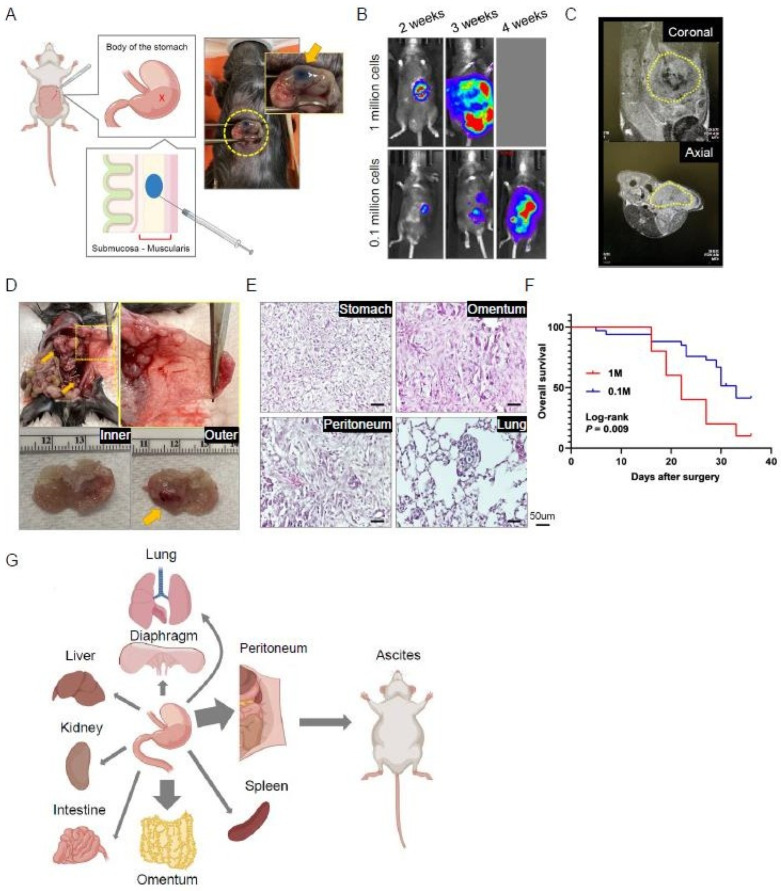
Procedure and characteristics of an orthotopic model of gastric cancer. Yellow cycles highlight the focus of the photos. (**A**) The illustration depicts the orthotopic injection of tumor cells into the stomach wall. (**B**) Representative bioluminescence images of injected mice. (**C**) Representative MRI images of injected mice taken 3 weeks after injection. (**D**) Representative macroscopic images of the abdominal cavity and peritoneal membrane of injected mice, as well as resected stomach and the developed primary tumor (arrow). (**E**) Representative hematoxylin and eosin (H&E)-stained images of tumoral tissues in multiple structures. (**F**) Survival curve of orthotopic mice injected with 0.1 million (*n* = 21) or 1 million (*n* = 12) tumor cells. (**G**) Graphical illustration of the orthotopic gastric cancer model and its progression into various organs.

Our usual injection method involves the submucosal injection of 0.1 million tumor cell suspensions with 10 μL PBS using a microsyringe that enables precise volume injection. It is crucial to ensure that there is only one bubble-like spot without any leakage on the stomach body wall after injection (Figure 2A). The tumor cells are supposed to be injected into submucosal and *muscularis propria* layers. We routinely monitor the tumor burden using a bioluminescence system, and after 2 weeks of injection, most mice show abdominal localized spotty signals (Figure 2B).

We have also recently implemented the use of an MRI image system, which allows for more precise visualization and quantification of the tumor (Figure 2C). At around 4 weeks post-injection, we can confirm the presence of tumors in the stomach wall, as well as metastases to adjacent organs, such as the omentum and peritoneal membranes (Figure 2D). Other metastases were also observed in the kidney, spleen, liver, and intestine (Figure 2E). Interestingly, lung metastases were confirmed in quite a few cases. This model also recapitulates ascites, with 35% of engraftment. We have previously tested two conditions for injection cell numbers—1 million vs. 0.1 million—and found that tumor burden and survival outcomes were significantly correlated with the injected cell number (Figure 2B,F). Our PDOX precisely reflects outcomes according to experimental conditions, and it also demonstrates high success rates of 96.7% for tumor development and solid reproducibility. Furthermore, our PDOX enables the recapitulation of tumor progression to adjacent organs and the development of ascites, which is similar to what is observed in real patients with GAC (Figure 2G). This standard model provides a platform for exploring various phenotypes in GAC research.

## 3. Challenges of the PDX Models

### 3.1. Heterogeneity Loss, Selection Bias, Clonal Evolution of Tumors and Stroma Replacement

Numerous studies have highlighted the usefulness of PDXs in cancer research, including basic and translational research, as well as preclinical and personalized medicine [2,3,4,9,19]. However, PDXs have limitations. The fidelity of cancer cells in the PDX models has been questioned due to heterogeneity loss, clonal evolution within the tumors, and selection bias for engraftment. As a result, early passage PDXs were thus recommended for in vivo studies and drug screenings [10]. Despite being presumed to represent the genomics of primary tumors, the analysis of 1110 PDX samples across 24 cancer types has revealed a rapid accumulation of gene copy number changes during PDX passaging, often due to the selection of preexisting minor clones. The copy number alterations (CNAs) acquired during PDX passages differed from those acquired during tumor evolution in patients, with several CNAs recurrently observed in primary tumors gradually disappearing in PDXs. This indicates that events undergoing positive selection in humans can become dispensable during propagation in mice. The genomic stability of PDXs was associated with their response to chemotherapy and targeted therapy, but the CNA landscapes of PDXs diverged substantially from those of their parental tumors during passaging. Therefore, it was concluded that genomic aberrations in PDXs are dynamic and continuous over passaging, and PDXs do not necessarily capture the genomic landscape of primary tumors better than cell lines, contrary to common belief [20].

While PDXs can broadly recapitulate the polygnomic architecture of human tumors, they do not fully account for heterogeneity in the TME. The presence and extent of pro and anti-tumor environments, including cancer-associated fibroblasts (CAFs) and tumor associated macrophages (TAMs), in the PDX models remain uncertain. Stromal and tissue architectures can significantly affect transcriptional regulation, but they are often overlooked in establishing PDXs [21]. Genetic heterogeneity within a tumor arises through clonal evolution, and patients with highly heterogeneous tumors are more likely to be resistant to therapy and have reduced survival. Clonal evolution also occurs during metastasis when a subset of cells leaves the primary tumor to form metastases, resulting in reduced genetic heterogeneity at the metastatic site. A bioinformatic approach, analyzing whole exome sequencing (WES) data from two breast cancer PDXs of metastases, revealed that the mouse stroma can be a confounding factor in assessing intra-tumor heterogeneity. However, orthotopic mammary pad engraftment can faithfully mimic the clonal evolution process in human patients during metastases [22]. To counter the aforementioned issues, several preventive strategies are recommended. Preserving the heterogeneity of primary tumors during PDX engraftment, ensuring consistent serial in vivo passages, and minimizing in vitro passages in petri dishes are crucial measures. Careful selection of tumor sources during sample collection, as well as considering a multi-site engraftment approach or an orthotopic model, can be effective practices to address these concerns. In this context, the biobanking of primary tumors and corresponding PDXs in repositories assumes increasing significance.

**Figure 3:**
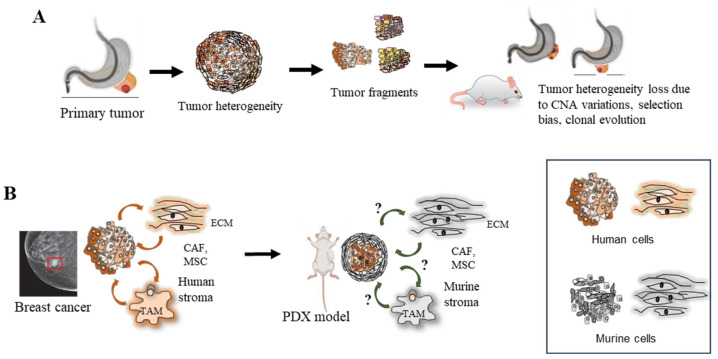
Clonal evolution and heterogeneity, as well as stromal environmental change, in PDX (modified from Shi et al., 2020, Ben-David et al., 2017, Sprouffske et al., 2020, and Cassidy et al., 2015) [10,20,21,22]. (**A**) Genomic aberrations in PDXs are dynamic and continuous over passaging, fidelity is lost due to CNA variations, selection bias, and clonal evolution. (**B**) Signaling pathways from stromal cells may not present to the same extent in PDX models as in primary tumors. The stromal environment can have profound effects on transcriptional regulation, but these are often overlooked in PDX models due to stromal replacement.

Figure 3A illustrates the phenomena of heterogeneity loss, selection bias, and clonal evolution in PDX models, while Figure 3B shows stromal changes reflected in tumor immune microenvironments, including CAFs and mesenchymal stem cells (MSCs).

### 3.2. The Lack of Immune Cells and Low Tumor Take Rate

Among the challenges of the current PDX models, one is their lack of immune cells in the TME compared to human tumors; in other word, they are lacking a complete immune system. Tumor take rates of PDXs are generally very low, as reported in breast cancers [15,23]. A study reported an overall take rate of only 27.4% of 113 breast cancer patient samples [11]. Tumors at advanced stages and with higher histological grades have a greater propensity to engraft successfully as PDX [17]. To overcome the low take rate in PDX models, the development of new immunodeficient mice and/or better tumor transplantation methods are currently recommended [15]. There is one such improvement that involves the addition of Matrigel to the injected cells, which has shown a statistically significant increase in the tumor engraftment rate in the colorectal carcinoma (CRC) PDX models [24].

To assess the significance of the human-to-murine stromal replacement for the fidelity of colorectal carcinoma (CRC) and its liver metastases in PDXs, a metabolic analysis was conducted between six patient tumors and corresponding PDXs across four generations. Although human stroma was entirely replaced at the second generation of PDX passages, the results showed that PDXs maintained functional stability at the metabolic level despite early replacement. The findings suggest that human cancer cells actively “educate” murine stromal cells during PDX development to adopt the human-like phenotype [25]. However, current PDX models have different tumor take rates and are not suitable for early-stage cancer studies [17].

### 3.3. Human and Host Viral Infections in PDXs

The presence of viral infections in PDXs has been reported, hindering further experimentation. Murine leukemia viruses, murine AIDS virus-related provirus, and murine endogenous retroviruses (mERVs) have been detected in PDXs of various cancer types. However, mERVs are expressed transiently and at low levels in fresh PDX-derived cell cultures. In addition, mERV integration into the genome of human cells is rare, making it unlikely to affect PDX-derived cell lines [26,27].

Virus-induced cell fusions, implicated in cancer progression, have been observed in various malignancies, including Burkitt lymphoma, Hodgkin lymphoma, and GAC [28]. PDXs derived from human cancer tissues, originally intended for research, have exhibited unexpected transformations into lymphomas, posing a challenge. Notably, a study revealed that, among 80 established PDXs, 26 (32.5%) transformed into lymphomas in NOD/SCID mice, with 23 of these being EBV-positive. Interestingly, PDXs from GAC primary tumors showed a notably higher rate (24/126, 19.0%) of lymphoma formation compared to PDXs from CRC primary tumors (1/43, 2.3%) [29]. Statistical analysis indicated a significant association between cancer type, inflammation in the parent tumor, and lymphomagenesis in PDXs. Detection of EBV infection and inflammation in primary tumors could potentially mitigate lymphoma development in PDXs [29,30]. These findings suggest a potential link between viral infections and malignancy development, highlighting the importance of addressing viral infections in the PDX models. EBV has been linked to a multitude of lymphomas and other types of malignancies, such as GAC. To mitigate this unintended lymphomagenesis in studying other cancer types, studies to develop anti-EBV vaccines are being conducted [30]; however, additional efforts are required to fully address this limitation.

### 3.4. Human-to-Host Oncogenic Transformation and Murine Contamination

**Figure 4:**
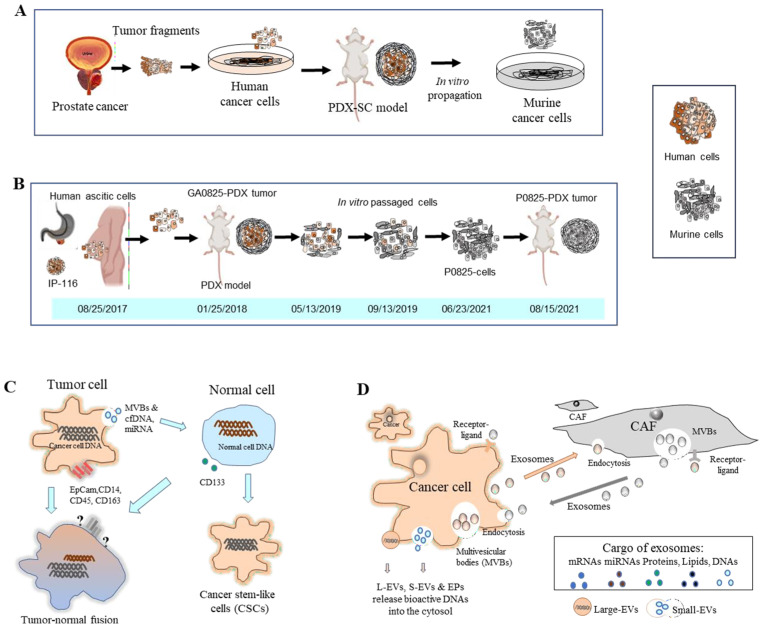
Human–mouse oncogenic transformation in a PDX model and hypotheses of tumor–normal cell oncogenic transformations. (**A**) In prostate cancer, human cells in PDXs were reportedly taken over by murine stromal cells (Adapted from Pathak et al. 1997, Ozen et al. 1997, and Multani et al. 1999) [32,33,34]. (**B**) In GAC PDX models, ascitic cells xenografted into a murine PDX turned murine stromal cells into oncogenic P0825 cells in a time-progressive manner (adapted from Jin et al., 2023) [31]. (**C**) Theories have been proposed regarding oncogenic transformations between tumor and normal cells. The tumor–normal cell fusion theory suggests merged DNAs, but some cancer markers may not be detected due to lost expressions. Another hypothesis is that cancer cells release cell-free DNAs (cfDNAs) and miRNAs to convert normal cells to cancer stem-like cells (adapted from Goldenberg 2012, 2013, 2014 [35,36,37]; García-Olmo et al., 2012 [38]; and Weiler and Dittmar 2019 [39]. (**D**) Crosstalks between cancer cells and stromal cells occur via exosomes that exchange through endocytosis, receptor–ligand interactions, and multivesicular bodies (MVBs). Cancer cells release bioactive DNAs into the cytosol via large extracellular vesicles (L-EVs), small EVs (S-EVs), and extracellular particles (EPs) (adapted from Fu et al., 2016, and Malkin and Bratman 2020 [40,41].

It is known that murine contamination is a widespread issue in cancer research labs. However, there are very limited reports on host contamination in cell lines and the PDX models. The Cytogenetics and Cell Authentication Core (CCAC) at M.D. Anderson Cancer Center found a contamination rate of host cells as high as 39% in cell lines sent for authentication [31]. Several studies demonstrated murine stromal and mixed human/murine cells in prostate cancer, indicating that human cancer cells cross-talked to murine stromal cells [32,33,34]. These observations indicated that human prostate tumors were transformed by human tumor cells into mouse oncogenic cells. We (Jin et al., 2023) reported that human ascitic cells (GA0825) from a GAC patient transformed murine stromal cells into a malignant tumorigenic murine P0825 cell line, in a PDX model, in a time-progressive manner [31]. Human-host oncogenic transformation in the PDXs is summarized in Figure 4A,B.

The mechanism of how human cancer cells transformed murine stromal cells in PDXs is not yet fully understood [32,33,34]. There are two hypotheses: [1] cell fusion and horizontal signal transfer or transmission and [2] the transfer of cell-free DNA (cfDNA). The former hypothesis is supported by studies that showed the in vivo fusion of human tumor cells with host cells and the horizontal transmission of malignant genes to host stromal cells. The latter hypothesis is supported by evidence that the TME includes microvesicles and that gene transfer, via the uptake of apoptotic bodies, may mediate the transformation of normal host cells.

Regarding the first hypothesis of human-host cell fusion and horizontal signal transfer or transmission, the in vivo fusion of the human tumor cells with hamster stromal cells and permanent transcription of human genes within were reported using human glioblastoma, lymphoma-hamster stromal cells [35,36]. Analyses using karyotyping, PCRs, and fluorescence in situ hybridization (FISH) proved that the spontaneous fusion of human tumors and host hamster cells occurred in vivo, and certain human chromosomes and genes were retained in the fused cells. Hence, it was hypothesized that cell fusion causes the horizontal transmission of malignant genes to host stromal cells. A hybrid tumor was found to have a total of 15 human chromosomes in its cells. Cancer cells can transform adjacent stromal cells, whose progeny permanently transcribe genes with malignant and other gene functions from the human donor DNAs. Using heterospecific in vivo cell fusion, genes encoding oncogenic and organogenic traits could be identified [37]. Now, accumulating evidence suggests that interactions between tumor cells and host cells in the TME are essential for tumor progression and metastases.

The transfer of cfDNA is the other potential hypothesis for cross-species transformation. Evidence suggests that microvesicles in the TME and the uptake of apoptotic bodies play a role in mediating the transformation of normal host cells. Additionally, plasma from CRC patients was able to transform cultured NIH-3T3 cells, and it generated carcinomas when injected into mice [38]. Another phenomenon is microvesicle-mediated signal transfer, which converts non-cancer stem cells into cancer stem cells through the activation of an ER^lo^/Notch^hi^ feed-forward loop, generating CD133^hi^ cancer stem-like cells [42]. The serum of cancer patients could induce oncogenic transformation of HEK293 cells in PDXs and maintain the self-renewal of hESCs (human embryonic stem cells), demonstrating the oncogenic transformation potential and the signal from cancer patient serum [43]. This tumor–normal cell oncogenic transformation is depicted in Figure 4C.

In our study of human ascites to murine stromal oncogenic transformation, no human–murine cell fusions were found, but a sole human somatic mutation, TP53-D281E, was found in the GA0825-PDX model by WES analysis [31]. This mutation may have played a role in oncogenic transformation, as it is adjacent to R282, one of the most common amino acid alterations in TP53 mutations that fail to bind to DNA, specifically, at the TP53 DNA regulatory sequence [44]. D281 is located at TP53’s regulatory contact area [45].

### 3.5. Murine Contamination and Host Carryover in Cell Lines and PDX Tumors

As mentioned above, cross-contamination of cell-lines and interspecies murine contamination in cell lines are widespread in research labs. To quantify murine cells in PDXs in lung cancer lines, genomic quantitation of murine stroma in PDXs was performed using human and murine species-specific PCR amplicon lengths (ssPAL). This method detected murine cell contamination, ranging from a few percentages to more than 95%, in lung adenocarcinoma and small cell lung carcinoma [46]. Murine contamination in cell lines, PDXs, and PDX-derived cells can affect preclinical drug-screening, parallel patient–animal in vivo studies, and laboratory in vitro experiments. A publication in 2019 reported pediatric neuroblastoma PDXs by using the TaqMan relative expression of mRNAs to differentiate human or murine tumors, yet the result was a yes or no for human or murine composition [47].

To quantify acute lymphoblastic leukemia (ALL) samples, a PDX Authentication System (PAS), combining an OpenArray assay of single nucleotide polymorphisms (SNPs), was developed to validate PDXs. This method detected 8 contaminated samples among 62 samples in a panel of PDXs from 73 leukemia patients. The identified SNP-discrepant PDXs demonstrated distinct gene expression profiles, indicating contamination [48]. A next-generation sequencing (NGS)-based method was able to quantify and authenticate human and murine cell lines, xenografts, and organoids with 0.1% sensitivity. This NGS method processes 100–200 samples in a single run, making it affordable [49]. To analyze host contamination, current software, such as Xenome, Disambiguate, Bamcmp, and pdxBlacklist, were compared to address contamination of murine DNAs and RNAs in PDX samples for the WES and RNAseq datasets [50]. The authors found that a major factor that can lead to incorrect mutation calls and the misidentification of canonical mutation signatures associated with tumorigenicity is incorrect identification of contaminating host reads when they analyzed DNAseq and RNAseq data from PDXs and circulating tumor cell-derived explant-derived WES and RNAseq data for NGS analyses [51]. Fluorescence-activated cell sorting (FACS) by flow cytometry is considered standard to separate and quantify human and murine cells in cells and PDXs. A method was developed for imaging cytometry using an EpCam marker integrated with the micropallet array technology to overcome host contamination in PDX-derived cells [52].

We (Jin et al., 2023) analyzed eight patients’ GAC ascites and their paired murine PDXs using so-called intronic genomic qPCR, and all the PDX tumors were found to carry different levels of murine stromal cells. There was one PDX that had a mix of 95.28% human and 4.72% mouse cells, while a second PDX had only 5.36% human cells and 94.64% murine cells. The latter PDX sample presents an obvious dilemma for in vivo drug treatment studies and preclinical personalized therapies if chosen for experiments [31].

### 3.6. Tumor Microenvironment (TME) within PDXs

In the context of PDXs, the TME has been recognized as an increasingly important topic due to cancer–host stroma cell crosstalk. The TME consists of cancer cells as well as a stroma of cellular and noncellular components. However, typically, anticancer therapies target cancer cells, and their effect on the tumor stroma is often not considered [53]. Furthermore, intratumoral heterogeneity can be influenced by tumor extrinsic factors in the TME, including murine host cells. After 3–5 passages, when PDXs can be used for drug screening, tumor-associated stroma is almost entirely replaced by a murine-derived extracellular matrix (ECM) and fibroblasts. This new murine stroma is likely to cause significant changes in the immunological regulation of the tumors and in physical properties [8]. The PDX models have also been used to investigate the various types of immune cells and stromal cells in the TME. CAFs and MSCs are local residents that influence cancer properties and surrounding TME. MSCs and TAMs are two representative cells in the TME that are “educated” by the TME. MSCs exert immunoregulatory effects on macro-phages and polarize to M2-like states via cell–cell contact and a paracrine or extracellular vesicle (EV) transfer mechanism [54]. These cells have been reported to facilitate tumor progression in studies with PDX tumors [54,55,56].

As for cancer–stroma interactions, exosomes shuffle macrovesicles from donor cells to target cells via endocytosis or multivesicular bodies (MVBs), which include proteins, messenger RNAs, miRNAs, nucleic acids, and lipids [40]. EVs and extracellular particles (EPs) have recently emerged as active carriers of molecular biomarkers and mediators of cell communication. The functional potential of EV/EP DNAs has been proposed in a number of pathological states, including malignancies and autoimmune diseases [41]. An illustration of cancer–stroma crosstalk is depicted in Figure 4D.

Although PDXs maintain the in vivo structure of tumors, human stromal cells gradually get replaced by murine counterparts after transplantation into immunodeficient mice. However, the implanted human cancer cells retain the potential to recruit murine stromal cells to their niche. Nevertheless, there are differences between the ligands secreted by human and murine fibroblasts. Human IL-2 stimulates the proliferation of murine T cells, whereas mouse IL-2 stimulates human T cells with significantly lower efficiency. T cell-stimulating IL-4 appears to be species-specific, and human NK cells are less sensitive to murine IL-15. Therefore, co-implantation of human CAFs and tumor cell suspensions, extracted from PDXs into secondary recipient mice, could provide an optimal setting for evaluating human tumor cell–stroma cell interactions [8]. For example, in a subset of patients with advanced Her2+ breast cancer, drug resistance develops after implementing multiple Her2-targeted therapies. In the TME, CAFs counteract the cytotoxic effects of Her2 kinase-targeted therapy in cancer cell lines and allow cancer cells to proliferate in the presence of the Her2 kinase inhibitor lapatinib [57].

## 4. Prospects and Overcoming limitation of PDXs

### 4.1. Organoids or 3D Culture

In recent years, three-dimensional (3D) in vitro models have been developed for pancreatic ductal adenocarcinomas. These models range from spheroids, scaffold models, and bi printed models to organ-on-chip models with the aim of maintaining the complexity and heterogeneity of pancreatic cancers [58]. Among them, tumor 3D organoids are widely used in preclinical drug evaluation, biomarker identification, biological research, and individualized therapy. Cancer organoids inherit the genomic and molecular characteristics of the donor tumor, providing a more individual model to predict the efficacy of anticancer treatment in vitro. The stability and fidelity of the cancer organoid drug screening model have been demonstrated in four aspects: ref. [1] results at different generations of organoids were mostly consistent; ref. [2] results of the tumor organoids were similar to the patients’ primary tumors; ref. [3] drug screening of the same organoid cell line was repeatable; ref. [4] results of organoid drug screening conform to previously reported genes and phenotypes [59,60,61]. While 3D organoids fall short of representing the human cancer milieu, co-culturing different cells mimicking the TME in complex 3D systems, based on cancer hallmarks, could potentially bridge this gap.

Patient-derived organoid culture is also a promising model for cancer research, preserving the key biological characteristics of the original tumor while reducing time and cost, as well as improving success rates compared to PDX models and cell lines [62]. Patient-derived cancer organoids provide a closer reflection of the pathophysiological features of natural tumorigenesis and metastases than conventional cell culture or PDXs. The technology has led to the development of patient-specific drug screening techniques, individualized treatment regimens, as well as the discovery of prognostic biomarkers and mechanisms of resistance. The combination of cancer organoids with other technologies, such as organ-on-a-chip, 3D bio-printing, and CRISPR/Cas9-mediated homology-independent organoid transgenesis, has shown promise in overcoming limitations [63,64,65,66]. Obviously, 3D organoids lose TME, which plays significant roles in crosstalk between tumors and CAFs, MSCs, ECM, and immune cells. Having stated that it should be acknowledged that 3D organoids fall short of representing the human cancer milieu, however, there is still a large gap between existing models and the ideal in vitro lung cancer models, and efforts to co-culture different cells to mimic the TME are ongoing [67].

### 4.2. Immunity and Humanized Mouse (HM) Models

HMs (Figure 1D) have been developed to overcome the limitation of PDXs in mimicking the interaction between cancer cells and immune cells in the TME. An HM is an immunodeficient mouse that is xenotransplanted with human cells or organs derived from fetal tissue or umbilical cord blood. This allows for the creation of human T cells, B cells, and other immune cells in mice, providing a more accurate reflection of the immune system of a human host [18].

Morton et al. (2020) have identified five major strains of HMs based on genetic modifications, categorized in Table 3, with each suited for different aspects of tumor biology research and patient response to immunotherapy [68]. HM PDX models are considered advantageous as they have a tumor immune environment closer to that of a patient’s TME. Despite the benefits, improvements in HMs are still needed, including the incomplete engraftment of immune cells, xeno-GvHD (graft-versus-host disease), and the lack of human cytokines and growth factors [28].

HM models provide a unique platform to evaluate the TME in vivo, particularly in assessing cancer treatments, including immune checkpoint inhibitors. To increase the responsiveness of human T cells to immunotherapies in HMs, strategies, such as T-cell education in a human thymus or the injection of previously educated T cells in human PBMCs into immunodeficient mice, have been proposed. While improvements are still needed, the use of HMs in tumor biology research and cancer treatment evaluation, particularly in assessing immune checkpoint inhibitors, holds significant potential [68].

### 4.3. Syngeneic Mouse Model

Technically, syngeneic mice (Figure 1E) are not PDXs, but they are gaining popularity for studying immunity, immune responses, and immunotherapy. Syngeneic mouse models are distinct from PDXs in that they do not use patient-derived cells or established human cancer cells; instead, the syngeneic models involve implanting tumors or tissues from the same species into immunocompetent mice, allowing for the study of the interaction between the immune system and tumor cells during tumor development and metastasis [69,70]. Syngeneic mouse models are particularly useful for testing immunologic drugs and investigating molecular or cellular manipulations on the immune system in immunocompetent recipient mice, which have the same genetic background as transplanted cells and TME. This model is designed to overcome the limitations of PDXs that lack a fully functional immune system, such as in nude, SCID, or NSG mice.

Preclinical studies on potential novel therapeutics for glioblastoma have been performed in immune-competent mice to identify immune-modulatory targets [71]. In cholangiocarcinoma (CCA), the syngeneic mouse models using murine cholangiocytes or hepatic organoids have been established in wild-type and immunodeficient mice to overcome the species mismatch between the tumors and the host animals [72]. Differences in genetic and cellular phenotypes have been identified between commonly used mouse syngeneic models and human cancers. The relative immunogenicity of these syngeneic tumors does not resemble typical human tumors derived from the same tissue of origin [73]. While results from syngeneic mouse models may not directly translate to humans, they may provide valuable proof-of-concept studies in a narrow context.

### 4.4. Detection and Quantification of Host Contamination in PDX Tumors and Cell Lines

As elaborated above, host contamination is not a negligible issue. In our research experience, murine contamination is widespread among human cancer lines, yet there are few publications in PubMed addressing this issue, nor is there a detection method that is fast, easy, and affordable. Murine contamination in cell lines, PDXs, and PDX-derived cells affects preclinical drug-screening, parallel patient, animal in vivo, and laboratory in vitro trials.

**Figure 5:**
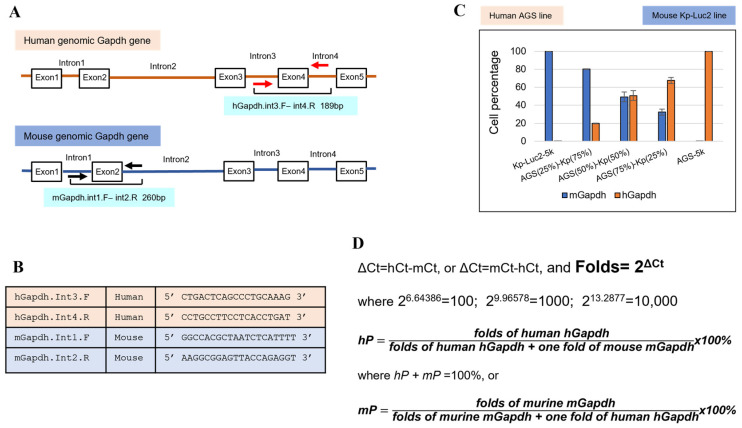
Intronic genomic qPCR for authenticating species, detecting and quantifying murine contamination in biosamples (Jin et al., 2023) [31]: (**A**) the human and murine intronic qPCR primers flank human Exon4 and murine Exon2 on the introns, respectively; (**B**) two sets of intronic genomic qPCR primers for human and mouse Gapdh, specifically (for SYBR Green qPCR); (**C**) an example of this intronic qPCR quantification using human AGS cells and murine Kp-Luc2 cells in standalone and mixed populations, showing a linear increment and decrement of human/murine cell numbers, which validates this intronic qPCR method; (**D**) the principle of copy number calculation (human percentage hP and murine percentage mP) in a biosample (a cell line or a PDX tumor) follows a similar fashion as in relative quantitation qPCR (folds = 2^Δct^).

Recently, we developed a novel method termed “intronic genomic qPCR” (Jin et al., 2023), which can authenticate and quantify human/mouse genomic copies with high sensitivity within a few hours. We analyzed eight malignant ascites of GAC patients and their paired murine PDXs, all of which carried different levels of murine stromal cells, as discussed in Section 3.5 [31]. The sensitivity of this method is either on par with or better than NGS analysis. A diagram of this intronic genomic qPCR is illustrated in Figure 5, with the major elements and methodology displayed in detail. Briefly, this qPCR method utilizes the SYBR Green technique to accurately detect human and/or murine genomic copies of the housekeeping *Gapdh* gene within the genomic DNAs of the samples, thereby avoiding mRNA or cDNA copies.

## 5. Conclusions

In this review, we discussed the PDX models as important preclinical models used in cancer research. We highlighted their merits as standard in vivo models for cancer research, but we also discussed various challenges faced by investigators. We also provided future insights and solutions into the trending orthotopic, 3D organoid culture, and interspecies authentication, detection, and quantification method, which is termed intronic genomic qPCR. Overall, the PDX models offer direct research on patient specimens, and researchers should be aware of the limitations and challenges associated with these models and continually work to develop new and improved techniques for cancer research.

## Figures and Tables

**Table 1:** The three most common types of PDX models for cancer research (modified from Byrne et. al. 2017) [11].

PDX Models	Advantages	Disadvantages
(1) Primary tumor specimens implanted subcutaneously (PDX-SC)	Intact primary tumor tissue and architecture Captures clonal diversity Easy to measure tumor responses Intravital tumor imaging	No proper anatomical niche Only suitable for higher grade, more aggressive tumors
(2) Primary tumor specimens implanted orthotopically (PDOX)	Intact primary tumor tissue and architecture Matching primary tumor in context Metastasis assessment from primary tumor Both primary and metastatic tumor niche Recapitulates metastatic process Ability to mimic clinical scenarios	Need extra technology to visualize tumor Challenging microsurgical skills Impossible for large collections and high throughput engraftment
(3) Humanized mouse (HM) models	Recapitulates human immune system in mice	Lengthy mouse humanization procedures Hurdles to achieve complete human immunity reconstitution

**Table 2:** Strains of mouse host types for PDX models, according to Shultz et al. (2007) [18].

Strain Name	Phenotype	Strain Name	Phenotype
C57BL/6-nu	Nude, athymic, lacks T cells	NOD-Rag1−/−	NOD+ Rag1 mutation leading to lack of mature T and B cells
CB17-SCID	Lacks mature T and B cells; radiation sensitive	NOD-Rag1−/− Prf1−/−	NOD+ Rag1 mutation leading to lack of mature T and B cells; lack of perforin
NOD- SCID	No mature T and B cells; radiation sensitive; decreased innate immunity	NOD-SCID HLA-A2.1-transgenic	NOD-SCID+ Transgenic expression of human HLA-A2.1
BALB/c-SCID bg	No mature T and B cells; radiation sensitive; decreased NK-cell activity	NOD/LtSz-SCID Il2rg−/−	No mature T and B cells; radiation sensitive; IL-2Rγ-chain deficiency; reduced multiple cytokine receptors thus many innate immune defects
C57BL/6-SCID bg	No mature T and B cells; decreased NK-cell activity	NOD/Shi-SCID Il2rg−/−	Similar to NOD/LtSz-SCID Il2rg−/− mice
NOD-SCID B2m−/−	No mature T and B cells; radiation sensitive; no β2m, leading to lack of MHC class I expression	BALB/c-Rag2−/− Il2rg−/−	Similar to NOD/LtSz-SCID Il2rg−/− mice
NOD-SCID IL-3-, GM-CSF and SCF transgenic	No mature T and B cells; radiation sensitive; transgenic human cytokine production	H2d -Rag2−/−Il2rg−/−	Similar to NOD/LtSz-SCID Il2rg−/− mice

**Table 3:** The five major HM strains and their attributes for cancer research, according to Morton et al. (2020) [68] *.

Mouse Model	Attributes of the Human Immune System
NSG+ hPBMCs	Adult T cells, educated in a human thymus, infiltrate implanted tumors but are alloreactive to the mouse
NSG+ cord blood HSCs	Murine T-cell education; incomplete development of B cells and myeloid cells
NSG+ fetal BLT	Human thymic education produces active T cells
BRG+ HSCs+ human cytokines	Greater human immune cell populations; improved myeloid cell maturation
hHLA-A * 02-NSG	T-cell education guided by a human antigen; improved T-cell activity

* hPBMCs: human peripheral blood mononuclear cells; HSCs: hematopoietic stem cells; BLT: bone marrow, liver, and thymus; HLA: human leukocyte antigen.

## Data Availability

The data presented in this study are available in this review.
